# A Bayesian network analysis of psychosocial risk and protective factors for suicidal ideation

**DOI:** 10.3389/fpubh.2023.1010264

**Published:** 2023-03-01

**Authors:** Jaime Delgadillo, Sanja Budimir, Michael Barkham, Elke Humer, Christoph Pieh, Thomas Probst

**Affiliations:** ^1^Clinical and Applied Psychology Unit, Department of Psychology, University of Sheffield, Sheffield, United Kingdom; ^2^Department for Psychosomatic Medicine and Psychotherapy, Danube University Krems, Krems an der Donau, Austria; ^3^Department of Work, Organization and Society, Ghent University, Ghent, Belgium

**Keywords:** depression, risk factors, Bayesian network analysis, suicide, COVID-19

## Abstract

**Background:**

The aim of this study was to investigate and model the interactions between a range of risk and protective factors for suicidal ideation using general population data collected during the critical phase of the COVID-19 pandemic.

**Methods:**

Bayesian network analyses were applied to cross-sectional data collected 1 month after the COVID-19 lockdown measures were implemented in Austria and the United Kingdom. In nationally representative samples (*n* = 1,005 Austria; *n* = 1,006 UK), sociodemographic features and a multi-domain battery of health, wellbeing and quality of life (QOL) measures were completed. Predictive accuracy was examined using the area under the curve (AUC) within-sample (country) and out-of-sample.

**Results:**

The AUC of the Bayesian network models were ≥ 0.84 within-sample and ≥0.79 out-of-sample, explaining close to 50% of variability in suicidal ideation. In total, 15 interrelated risk and protective factors were identified. Seven of these factors were replicated in both countries: depressive symptoms, loneliness, anxiety symptoms, self-efficacy, resilience, QOL physical health, and QOL living environment.

**Conclusions:**

Bayesian network models had high predictive accuracy. Several psychosocial risk and protective factors have complex interrelationships that influence suicidal ideation. It is possible to predict suicidal risk with high accuracy using this information.

## Background

Suicide is a serious global health problem, with an age-adjusted annual global incidence rate of 11.4 per 100,000 (1). Suicide represents the leading cause of death worldwide among young people, disproportionately affecting males living in environments with high economic inequalities (2). There are indications that for every suicide there are over 20 times more people who attempt suicide (1). This already alarming situation may have been further aggravated by the outbreak of the novel coronavirus disease (COVID-19). The COVID-19 outbreak has dramatically impacted health, economics and social connections around the world (3), thereby exacerbating known risk factors for suicidal ideation and suicide attempts (4, 5). These risk factors include forced isolation, quarantine, reduction of social contacts, health-related anxiety, economic problems, risk of domestic violence, risk of addictive behavior and reduction of access to mental health care (6). The COVID-19 pandemic might lead to an increase in rates of self-injury or suicide, especially in individuals with pre-existing mental health problems (i.e., depression or anxiety), but also in people under increased stress such health care professionals (6–8). Therefore, this public health emergency calls for advances in suicide research and prevention (7).

Understanding suicide risk is crucial in order to advance the implementation of effective prevention strategies. Traditional attempts at understanding the antecedents of suicide have focused on single risk factors, or a specific domain of risk (i.e., socio-demographics), and thus have been of limited value to the design of effective prevention measures (9). Literature in this field has identified some risk factors such as genetic and biological factors, mental disorders, and stressors such as financial problems or violence (1, 10, 11). However, risk prediction accuracy is still limited due to the low explained variance afforded by these variables (9). Low base-rate events such as suicide are also notoriously difficult to predict, which limits the reliability of risk factor research. Furthermore, the complexity of factors leading to suicidal behavior cannot be adequately addressed by conventional statistical techniques, such as regression analysis or analysis of variance, as they provide limited insight into the interrelationships between the risk factors themselves (12).

Compared to actual suicide attempts, suicidal ideation, which refers to thoughts of engaging in behavior intended to end one's life, is more than three times more prevalent in the general population (13). In this regard, studying suicidal ideation as a proximal antecedent to suicidal behavior could offer a way forward in understanding key risk and protective factors, and enable the development of *just in time adaptive interventions* (14, 15). However, the real-time monitoring of risk factors involves considerable participant burden. Accordingly, deploying these assessments at a population-scale, and even in clinical samples, seems unfeasible (14). A more realistic strategy could be to deploy such interventions in a targeted way, focusing on people at *high risk* of suicide. As the ability to predict suicide risk has not improved in the past 50 years (9), it is necessary to investigate the combined effects of multiple factors to characterize this *high risk* phenotype with greater precision. However, so far the study of factors contributing to suicidal thoughts have rarely examined the combined effect of multiple risk factors and protective factors. Also, large data sets including multiple potential risk and protective factors are required to enable reliable prediction research (9, 16).

Methodological developments such as machine learning and network analysis represent a novel way to predict health-related outcomes and to model complex interrelationships between variables in a causal network (17, 18). Unlike conventional hypothesis-testing studies that specify expected relationships *a priori*; machine learning offers an exploratory and data-driven framework to discover patterns of associations in large datasets. Conventional approaches to model risk factors for suicidal ideation tend to focus on the statistical significance and explained variance attributable to specific variables that are selected based on prior theory or research (i.e., main effects for hypothesized predictors). Machine learning analyses are not necessarily constrained to the modeling of main effects, and can “discover” complex (i.e., non-linear, interactive) relationships between variables, which were not previously known or expected. Rather than prioritizing goodness-of-fit in a single dataset as in conventional regression analysis, machine learning frameworks use cross-validation methods to determine if discovered relationships in the data have adequate predictive accuracy, and are therefore potentially generalizable to new samples. As such, network analysis of suicidal ideation and its risk and protective factors could potentially help to derive new insights in the field of suicide prevention (12). The present paper aims to contribute to the identification of reliable risk and protective factors for suicidal ideation from a data-driven perspective, without prior specification of hypotheses, but using variables that have been selected based on prior evidence described above. To this end, we developed Bayesian network models using data from a cross-sectional survey conducted during the peak of the first COVID-19 lockdown in two European countries, Austria and the United Kingdom.

## Methods

### Design and setting

The objectives of the present study were (1) to identify predictors of suicidal ideation (2), to model complex interactions between these predictors, and (3) to examine their generalizability across two countries. We approached this from a machine learning perspective, using a cross-country cross-validation design to enable us to understand which predictors replicate in samples from two different countries. A cross-sectional online survey was designed to recruit representative samples covering all geographical regions of Austria and the United Kingdom (UK), and reflecting population norms in relation to demographic features. The Qualtrics^®^ population survey platform was used; implementing age, gender, educational, and regional quotas based on available population census data from both countries. The survey measured sociodemographic features and several health, wellbeing and quality of life indicators that were informed by prior evidence. Data collection started 4 weeks after COVID-19 lockdown measures were implemented in Austria and the UK (April 2020), until the point where a representative sample was obtained with a minimum sample size of *n* = 1,000 participants from each country, which was specified *a priori*. Participants were recruited from existing pools of research panel participants and received financial incentives. Participants who did not respond to all questions or who failed quality checks, including attention filters and survey timings, were excluded. The goal of the sampling procedure was to obtain large enough samples from each country in order to conduct machine learning analyses, which were nationally representative (covering all regions of each country in a proportionate way, reflective of local demographics), and balanced between both countries (same sample size, to minimize imbalance due to differences in overall population density across countries). Overall, the target sample was attained within 10 days, after which the survey closed.

### Measures

The primary outcome of interest was suicidal ideation, derived from the Patient Health Questionnaire (PHQ-9), which has been shown to be a robust and age-independent predictor of suicide attempts and deaths (19). The PHQ-9 is a measure of depression symptoms, where response options for each of 9 questions are “not at all” (0 points), “several days” (1 point), “more than half of the days” (2 points) or “nearly every day” (3 points), yielding an overall severity score between 0 and 27 (20). A cut-off score of ≥10 has been recommended to screen for clinically significant depression symptoms, with adequate sensitivity (88%) and specificity (88%). Item 9 of the PHQ-9 measure asks, “Over the last 2 weeks, how often have you been bothered by thoughts that you would be better off dead or of hurting yourself in some way?” Response to this question was coded in a binary way to identify the presence of any recent suicidal ideas within the last 2 weeks (1 = item endorsed if response ranged from 1 to 3; 0 = item not endorsed if response was 0). The remaining items (PHQ-8) were used to control for depression severity (21).

#### Health and wellbeing indicators

The GAD-7 is a 7-item case-finding measure for anxiety disorders; each item is rated between 0 and 3, with a total severity score between 0 and 21 (22). Stress-severity was measured with the PSS-10, which measures two related domains (perceived helplessness, perceived self-efficacy) using 10 items on a five-point scale ranging from 0-4 (23). The Insomnia Severity Index (ISI) (24) is a measure of sleep quality and insomnia, based on 7 items rated on a five-point scale (from 0 to 4). The WHOQOL-BREF is a 26-item questionnaire that measures four domains of quality-of-life; physical health, psychological health, social relationships, and environment, during the past 2 weeks (25). Social loneliness was measured using the 11-item De Jong-Gierveld scale (26). Resilience was assessed using the 10-item version of the Connor-Davidson resilience scale (CD-RISC-10) (27), where items are rated using a Likert scale from 0 to 4. Single-item questions were used to assess self-reported days of exercise per week and physical illness status.

#### Demographics

Participants completed single-item questions to gather the following demographic features: age, gender, highest level of education, marital status, having children requiring care, employment status, net household income, housing type, household number of occupants additional to the respondent.

### Statistical analysis

**Figure 1:**
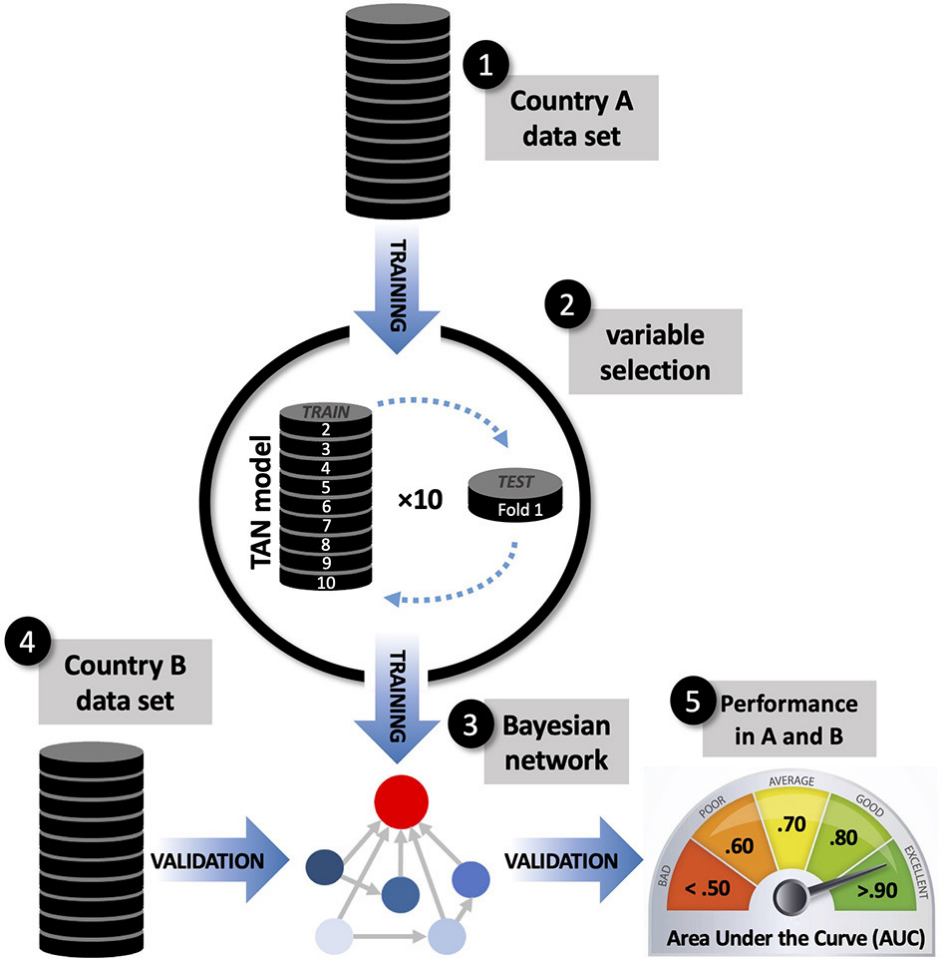
Schematic representation of the cross-country, cross-validation design used to identify suicidal ideation risk factors that are country-specific and those that are common across samples. Model training used a tree-augmented naïve Bayes algorithm, applying 10-fold internal cross-validation for variable selection. Classification accuracy was assessed within-sample and out-of-sample using the Area Under the Curve (AUC).

A cross-country, cross-validation design was used to identify suicidal risk factors that may be country-specific and those that are common across samples. This design, depicted in Figure 1, involved training a prediction model for each country and then testing its generalizability using data from the other country to classify cases as belonging to the suicidal or non-suicidal class.

Each country-specific prediction model was trained using a Tree-Augmented Naïve Bayes (TAN) algorithm (18). Unlike conventional multivariable logistic regression models which only model main effects, or require pre-specification of expected interactions, the TAN method offers a data-driven way to model a network of relationships (called *attribute dependencies*) between predictors and their joint influence over a target outcome. TAN produces a simple and parsimonious network model where each predictor is allowed to depend on one additional predictor, thus modeling multiple two-way interactions. The risk of suicidal ideation is thus estimated based on the combined weight (e.g., joint modeling) of the conditional probabilities attributed to each predictor in a Bayesian network model.

Like other machine learning approaches, the performance of the TAN algorithm depends largely on the adequacy of variable selection. In order to build Bayesian networks composed only with reliable predictors, we entered all available variables listed above in the Measures section and performed variable selection using a ten-fold cross-validation (CV10) approach (28). Two noise variables (continuous and categorical) were modeled on the distribution of PHQ-9 (mean, standard deviation) and gender (base rate of males and females). Noise variables were introduced as predictors in the TAN analysis, along with all other candidate predictors listed in Table 1. The CV10 approach produced ten Bayesian network models (within each country-specific sample) with their respective variable importance plots, which ranked candidate variables according to their predictive value. We performed variable selection by only retaining the variables that were consistently ranked as more important than both noise variables in more than half (>5) of the trained models. The selected variables were entered into a final country-specific Bayesian network model, which was visualized using a directed acyclic graph (29). The CV10 procedure was strictly used for variable selection and not for hyperparameter tuning. TAN was applied using pre-specified hyperparameters (using likelihood ratio as the independence test; significance level of 0.01; maximum conditional set size = 5; using Bayes adjustment for small cell counts).

**Table 1:** Sample characteristics.

	**Austria (*N* = 1,005)**	**United Kingdom (*N* = 1,006)**
**Demographics**		
Age group, % (*n*)		
18–24	11.7 (118)	9.7 (98)
25–34	16.5 (166)	20.2 (203)
35–44	18.4 (185)	18.9 (190)
45–54	22.1 (222)	19.3 (194)
55–64	18.0 (181)	17.2 (173)
65+	13.2 (133)	14.7 (148)
Females, % (*n*)	52.7(530)	54.1 (544)
Education, % (*n*)		
None at all	0.0 (0)	1.6 (16)
Elementary school	0.10 (1)	3.5 (35)
High school	2.6 (26)	40.3 (405)
Vocational training	31.9 (321)	14.2 (143)
College degree	28.7 (288)	12.8 (129)
University degree	36.7 (369)	27.6 (278)
Children, % (*n*)	23.7 (238)	30.6 (308)
Employment status, % (*n*)		
Unemployed	26.8 (269)	47.5 (478)
Employed	55.8 (561)	38.5 (387)
Retired	17.4 (175)	14.0 (141)
Household income, % (*n*)		
Band 1	7.1 (71)	13.7 (138)
Band 2	23.4 (235)	34.1 (343)
Band 3	30.2 (304)	25.4 (256)
Band 4	19.5 (196)	14.6 (147)
Band 5	19.8 (199)	12.1 (122)
Housing type, % (*n*)		
Flat	23.2 (233)	20.1 (202)
Apartment with terrace	34.4 (346)	5.6 (56)
House	42.4 (426)	74.4 (748)
Household occupants, mean (SD)	1.74 (1.34)	1.89 (1.43)
**Health and wellbeing**		
Illness reported, % (*n*)	6.9 (69)	10.3 (104)
Days exercise per week, mean (SD)	2.70 (1.44)	2.29 (1.59)
Suicidal ideas, % (*n*)	17.3 (174)	31.7 (319)
Suicidal ideas with depression^*^, % (*n*)	55.0 (553)	64.5 (649)
Suicidal ideas without depression^*^, % (*n*)	7.3 (73)	8.8 (89)
PHQ-8, mean (SD)	5.93 (5.00)	8.38 (6.99)
GAD-7, mean (SD)	5.84 (4.70)	8.03 (6.52)
PSS10 helplessness, mean (SD)	9.37 (5.19)	10.34 (6.05)
PSS10 self-efficacy, mean (SD)	9.40 (3.13)	8.63 (3.42)
Insomnia severity index, mean (SD)	8.31 (5.70)	10.43 (7.05)
Loneliness scale, mean (SD)	4.58 (3.67)	6.41 (3.21)
CD-RISC-10, mean (SD)	27.27 (7.20)	24.56 (8.12)
WHOQOL physical, mean (SD)	15.57 (2.77)	14.58 (3.31)
WHOQOL psychological, mean (SD)	15.17 (2.99)	13.38 (3.42)
WHOQOL relationships, mean (SD)	14.41 (3.47)	13.67 (3.83)
WHOQOL environment, mean (SD)	15.96 (2.43)	14.35 (2.97)

*n*, frequencies; SD, standard deviation; Monthly household income bands = Austria (< €1k, €1k to €2k, €2k to €3k, €3k to €4k, >€4k), UK (< £900, £900–1,800, £1,800–2,700, £2,700–3,600, >£3,600); PHQ-8, depression severity measure excluding suicidal ideation; GAD-7, anxiety severity; CD-RISC-10, resilience; WHOQOL, quality of life across four domains.

* depression status is based on PHQ-9 ≥ 10.

Once the country-specific Bayesian network models were trained, we calculated their explained variance based on Nagelkerke R^2^. Classification accuracy was assessed within-sample and out-of-sample using the area under the curve (AUC), positive and negative predictive values (PPV, NPV).

## Results

### Sample characteristics

Sample characteristics for the Austrian (*n* = 1,005) and UK (*n* = 1,006) samples are displayed in Table 1. The prevalence of suicidal ideation was higher in the UK sample (31.7%) compared to the Austrian sample (17.3%). As expected, participants with clinically significant depression symptoms (PHQ-9 ≥ 10) tended to have a high prevalence of suicidal ideas (Austria = 55.0%; UK = 64.5%). However, around 8% of non-depressed participants also endorsed suicidal ideas, indicating that other risk factors may also be relevant.

**Figure 2:**
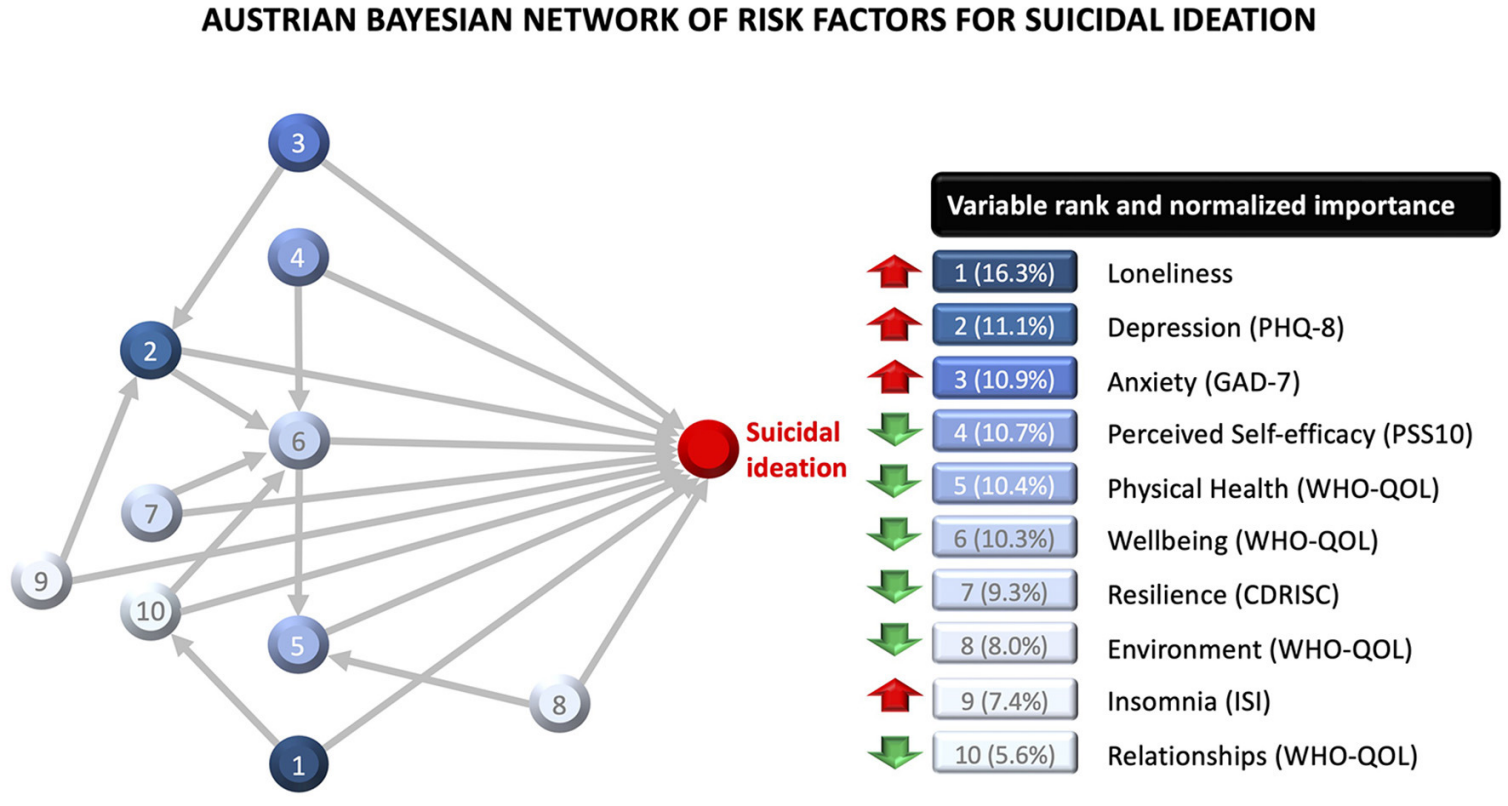
Bayesian network model for the Austrian sample, with variable importance indices for each variable. The red upward arrows denote risk factors for suicidal ideation, and the green downward arrows denote protective factors. The model also shows two-way interactions between variables.

**Figure 3:**
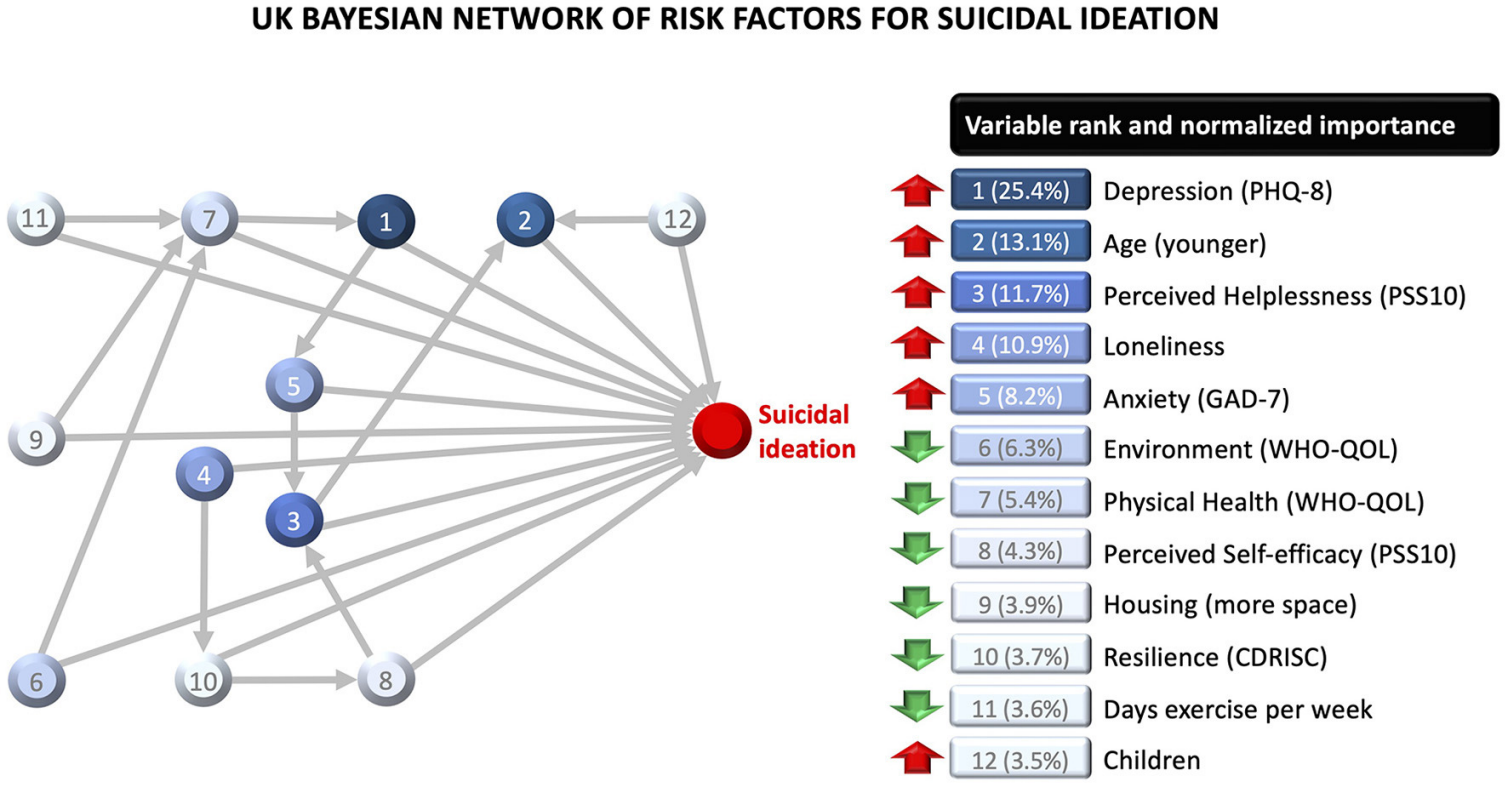
Bayesian network model for the British sample, with variable importance indices for each variable. The red upward arrows denote risk factors for suicidal ideation, and the green downward arrows denote protective factors. The model also shows two-way interactions between variables.

Country-specific Bayesian network models are presented in Figures 2 and 3, along with normalized (0–100%) variable importance indices that quantify each variable's contribution to explained variance. The upward red arrows denote factors that increase risk of suicidal ideation, and the downward green arrows denote protective factors. The model also shows inter-relationships between the variables.

The Austrian model included ten variables, of which the five most important ones were loneliness, depression, anxiety, perceived self-efficacy, and quality of life related to physical health. The model also showed multiple inter-relationships between the factors. The effect of wellbeing was moderated by self-efficacy, depression, resilience, and quality of relationships. The effect of depression was moderated by anxiety and insomnia. The effect of relationship quality was moderated by loneliness. The effect of physical health was moderated by quality of the environment. Overall, this network model explained 47.1% of variability in suicidal ideation in the Austrian sample. The model's classification accuracy was similar within-sample (AUC = 0.84; PPV = 0.69; NPV = 0.94) and out-of-sample (AUC = 0.80; PPV = 0.61; NPV = 0.83), with minimal prediction shrinkage (AUC = 0.04).

The UK model included 12 variables, of which the five most important ones were depression, age, perceived helplessness, loneliness and anxiety. All variables interacted with other variables in the network. The effect of physical health was moderated by exercise, housing space, and quality of the environment. The effect of helplessness was moderated by anxiety and self-efficacy. The effect of resilience was moderated by loneliness. The effect of anxiety was moderated by depression, which in turn was moderated by physical health. The effect of age was moderated by helplessness and having children requiring care. Younger parents were at increased risk of suicidal ideation relative to younger people without children requiring care; but older parents (≥45) were at reduced risk compared to older people without children requiring care. Overall, this network model explained 49.5% of variability in suicidal ideation in the UK sample. The model's classification accuracy was better within-sample (AUC = 0.93; PPV = 0.75; NPV = 0.90) than out-of-sample (AUC = 0.79; PPV = 0.51; NPV = 0.91), with a prediction shrinkage of AUC = 0.14.

## Discussion

Using large and representative samples from two European countries, this study identified psychosocial risk and protective factors for suicidal ideation during the acute phase of the COVID-19 lockdown. Fifteen relevant factors were identified, of which seven were replicated in both countries: depression, loneliness, anxiety, self-efficacy, resilience, and quality of life related to physical health and the living environment. These results are consistent with evidence from prior meta-analyses and systematic reviews focusing on mood disorders (13, 30, 31), loneliness (32), and poor physical health (33), which are well-known risk factors for suicidal thoughts and behavior. Similarly, self-efficacy (34) and resilience (12) have been found to be inversely related to suicide ideation as supported by the present findings.

The COVID-19 pandemic might have exacerbated the impact of some of these risk factors. For example, the significant negative consequences of isolation and social distancing might increase loneliness (35), which was found to range among the most important risk factors in both countries, explaining 16.3% of variability in suicidal ideation in the Austrian sample and 10.9% in the UK sample. Depression and anxiety, which also ranged among the five most important factors for suicidal ideation in both countries, were also found to significantly increase during the COVID-19 lockdown as compared to previous epidemiological data (36, 37).

The substantial effects of the COVID-19 pandemic on the global economy have been predicted to cause an increase in suicides related to an increase in the unemployment rate of about 2,135 (low scenario) to 9,570 (high scenario) per year (5). Also a narrative historical paper examining how previous disasters (natural disasters, violence, war, epidemics/pandemics, and economic recession) affected suicidal behavior, found that among all the types of disasters, economic recession had the most significant impact on suicide rates (38). Contrary to these studies, the current analysis revealed no association between employment status or net household income and risk of suicidal ideation in Austria as well as UK. However, the downsizing of the economy might lead to unintended long-term problems if unemployment rates rise. Therefore, results might differ from the time during the COVID-19 lockdown or some weeks/months later, as unemployment rates might increase with time, which might also cause a change in the relationship of employment status and income with suicidal ideation.

A direct comparison of the prevalence of suicidal thoughts (17.3% in Austria, 31.7% in UK) with pre-pandemic values is not possible due to a lack of comparable data. However, in the UK face-to-face interviews conducted in 2014 revealed that 5.4% of 16–74 year old participants experienced suicidal thoughts in the past year (39). Even a recent study conducted in outpatients treated for mental disorders did not report suicidal thoughts over the last 2 weeks in the majority (80%) of the patients using the same measure of suicidal thoughts as we did (19). Therefore, it can be assumed that the situation around the COVID-19 pandemic considerably increased suicidal thoughts in the general population, with more than a 1.8-fold higher prevalence in the UK compared to Austria. One explanation for the higher prevalence in the UK might be that the UK was more badly affected by the pandemic than Austria. According to available information from the World Health Organization (WHO), the UK was among the most affected countries in Europe with the highest death rates at the time of the COVID-19 lockdown, while Austria was among the less affected countries. At the time of the start of the online survey, the cumulative number of confirmed deaths related to the COVID-19 pandemic was 28.6 per 100,000 population in UK compared to 3.3 deaths per 100,000 population in Austria (40, 41). However, further studies are required to reveal the underlying causes in the different prevalence rates of suicidal ideation. A number of culture-specific differences between both countries exist. For instance, the mental healthcare system is organized differently in both countries. While in the UK mental health care is widely available through the National Health Service (NHS), providing free of charge mental health services for individuals who are eligible for it (42), in Austria no general agreement covering psychotherapeutic care by national health services or social insurance institutions exists, with only a small fraction of all patients receiving a full refund of treatment costs, while the majority receives a small subsidization and funds their psychotherapeutic treatment themselves (43).

Furthermore, distinctive risk factors were identified in each country, providing evidence that suicidality is also influenced by culturally specific factors. For example, in the Austrian sample, insomnia increased risk, whereas psychological wellbeing and quality of relationships were protective factors. In the UK sample, suicidality was influenced by age, housing space, children requiring care, exercise and perceived helplessness. The application of Bayesian network models enabled the discovery of complex interrelationships between protective and risk factors. Observed interactions indicate that suicidality is influenced by an interplay of relational (parenthood, loneliness, quality of relationships), health indicators (physical health, depression, anxiety, exercise) and living conditions (housing space, quality of the environment). Of note, the effect of physical health was moderated by quality of the living environment in both countries. This fits with wider evidence that people living in socioeconomically deprived neighborhoods tend to have poorer overall physical and mental health (44–46), and with the notion that adverse life circumstances can lead to a sense of defeat and entrapment—as posited by the *integrated motivational–volitional model* of suicide (47). Furthermore, the important role of loneliness in the networks modeled in both countries is also consistent with contemporary theories such as the *interpersonal theory* (48) and the *three-step theory* of suicide (49). These results support the notion that the pathways to suicide ideation are complex, resulting from an interplay between several risk and protective factors (47, 50). A more precise understanding of the interrelations between key risk and protective factors can advance our efforts to rapidly identify people “at risk” of suicide, and to intervene early enough to prevent a transition from ideation to action, which is a central goal of most theories related to suicide prevention (51).

Aside from enabling the discovery of complex relationships among variables, the Bayesian network models had high predictive accuracy, explaining close to 50% of variability in suicidal ideation, which is a major improvement in terms of prognostic assessment and the identification of “at risk” cases. Furthermore, the variable importance indices displayed in Figures 1 and 2 demonstrate that this predictive value is not mainly driven by well-known risk factors such as depression and anxiety severity. In fact, depression and anxiety accounted for 22.0% (Austria) to 33.6% (UK) of the predictive value of the full network model. Classification accuracy was good (AUC .84) to excellent (AUC .93) within-sample, according to conventional standards in clinical medicine (52). The Austrian network model generalized impressively well to the UK sample, with minimal prediction shrinkage, since it was less complex and the majority of its predictors were common across countries. Higher out-of-sample prediction shrinkage was observed for the UK model, since it had a greater number of predictors that were country-specific. Overall, this cross-country prediction analysis indicates that the features contained in the more parsimonious of the two network models (Austria) has impressive generalizability to cases from a different sample and geographical region.

### Strengths and limitations

The major strengths of the study are the large, representative sample sizes and the cross-country cross-validation design. The conduct of the study in two countries, which were affected differently by the COVID-19 pandemic, allowed the investigation of the generalizability of predictors of suicidal ideation across countries. A further strength is the extensive battery of psychosocial variables and the application of machine learning approaches, enabling the modeling of interrelationships between several factors in a data-driven way. However, whether these high accuracies can be maintained or not in a non-pandemic context with lower base rates of suicidal ideation needs to be evaluated in further studies.

One major limitation of the study is its cross-sectional design, which does not allow a clear elucidation of the direction of the identified relationships, as suicidal ideation and behavior likely follow a cyclical nature (47). As no longitudinal assessments of the different risk and protective factors were conducted, this study was also not able to capture potential dynamics of changes in risk and protection states (14). A further limitation is that the network analysis applied in this study is only able to reveal two-way interactions between variables, whereas associations between three or more variables were not modeled. Furthermore, only self-ratings were used in the current study and clinician assessments were not applied, which might overestimate prevalence as people are often biased when they report their own experiences (53).

### Conclusions

Suicidal ideation can be accurately predicted using data from multiple risk and protective factors. Some of these factors were replicated across different countries, which is indicative of generalizability. The adverse consequences of the COVID-19 pandemic on increased depressive and anxiety symptoms, loneliness, and their strong connection to risk of suicidal ideation highlight the need to take urgent steps to prevent increased suicide rates during as well as in the aftermath of the COVID-19 pandemic.

## Data availability statement

The raw data supporting the conclusions of this article will be made available by the authors, without undue reservation. Data requests should be addressed in writing to TP (Thomas.Probst@donau-uni.ac.at).

## Ethics statement

This study involving human participants was reviewed and approved by an Independent Research Ethics Committee at Danube University Krems. Informed consent was obtained from all study participants.

## Author contributions

CP and TP were joint principal investigators and responsible for the design and conduct of the study. SB and EH supported survey design, data collection, and preparation. JD conducted data analysis. All authors contributed to the interpretation of results, writing, editing, and approval of the manuscript.
